# Transcriptome Analyses in Normal Prostate Epithelial Cells Exposed to Low-Dose Cadmium: Oncogenic and Immunomodulations Involving the Action of Tumor Necrosis Factor

**DOI:** 10.1289/ehp.11215

**Published:** 2008-03-03

**Authors:** Shlomo Bakshi, Xiang Zhang, Sonia Godoy-Tundidor, Robert Yuk Sing Cheng, Maureen A. Sartor, Mario Medvedovic, Shuk-Mei Ho

**Affiliations:** 1 Division of Environmental Genetics and Molecular Toxicology, Department of Environmental Health, and; 2 Cancer Center, University of Cincinnati College of Medicine, Cincinnati, Ohio, USA; 3 Center for Environmental Genetics, and; 4 Division of Biostatistics and Epidemiology, Department of Environmental Health, University of Cincinnati College of Medicine, Cincinnati, Ohio, USA

**Keywords:** carcinogenesis, cytokine, global expression profiling, heavy metals, immune response, inflammation, Ingenuity Pathway Analysis, knowledge-based analysis, prostate cancer

## Abstract

**Background:**

Cadmium is implicated in prostate carcinogenesis, but its oncogenic action remains unclear.

**Objectives:**

In this study we aimed to decipher changes in cell growth and the transcriptome in an immortalized human normal prostate epithelial cell line (NPrEC) following exposure to low-dose Cd.

**Methods:**

Synchronized NPrEC cells were exposed to different doses of Cd and assayed for cell viability and cell-cycle progression. We investigated changes in transcriptome by global profiling and used Ingenuity Pathways Analysis software to develop propositions about functional connections among differentially expressed genes. A neutralizing antibody was used to negate the effect of Cd-induced up-regulation of tumor necrosis factor (*TNF*) in NPrEC cells.

**Results:**

Exposure of NPrEC to 2.5 μM Cd enhanced cell viability and accelerated cell-cycle progression. Global expression profiling identified 48 genes that exhibited ≥ 1.5-fold changes in expression after 4, 8, 16, and 32 hr of Cd treatment. Pathway analyses inferred a functional connection among 35 of these genes in one major network, with *TNF* as the most prominent node. Fourteen of the 35 genes are related to *TNF*, and 11 exhibited an average of > 2-fold changes in gene expression. Real-time reverse transcriptase-polymerase chain reaction confirmed the up-regulation of 7 of the 11 genes (*ADAM8*, *EDN1*, *IL8*, *IL24*, *IL13RA2*, *COX2*/*PTGS2*, and *SERPINB2*) and uncovered a 28-fold transient increase in *TNF* expression in Cd-treated NPrEC cells. A TNF-neutralizing antibody effectively blocked Cd-induced elevations in the expression of these genes.

**Conclusions:**

Noncytotoxic, low-dose Cd has growth-promoting effects on NPrEC cells and induces transient overexpression of TNF, leading to up-regulation of genes with oncogenic and immunomodulation functions.

Prostate cancer (PCa) is currently the most common cancer in men, accounting for 29% of all new cases of cancer (Jemal et al. 2007). The etiology of PCa in humans is complex and may include age, race, and environmental and lifestyle risk factors, among other factors. The environmental factors such as exposure to cadmium that contribute to this disease have been less well studied than the aforementioned factors. Yet, there is epidemiologic and experimental evidence for a potential association between Cd exposure and PCa in humans and rodents (Goyer et al. 2004; Vinceti et al. 2007; Waalkes et al. 1989).

Cd is classified as a human carcinogen by the International Agency for Research on Cancer (IARC 1993) and the National Toxicology Program (NTP 2004). It is ubiquitously present in the environment because of industrial and other types of pollution. Occupational exposures are related to handling of waste associated with mining, smelting, electroplating, and manufacturing of batteries, pigments, and plastics (IARC 1993; NTP 2004). In contaminated areas, Cd permeates the soil and water supply, reaching levels as high as 0.21 mg/kg and approximately 1.9 μM in foodstuffs (Yan et al. 2007). Smokers are also exposed to Cd from cigarettes. In general, the main route of Cd exposure among nonsmokers is food intake (Satarug and Moore 2004). Human exposure is of serious concern in fast-developing countries such as China and India that have less stringent regulations (Govil et alet al. 2007; Sun et al. 2006; Yan et al. 2007). In humans, Cd is associated with lung cancer but is not definitively linked to PCa, although Cd can induce PCa in rodents (Waalkes et al. 1989). Because of its very slow excretion rates (~ 0.001%/day) (Satarug and Moore 2004), Cd accumulates in men as they age (Baecklund et al. 1999), and the prostate is one of the organs of the body with high bioaccumulation of Cd (0.45–28 μM) (Achanzar et al. 2001; Lindegaard et al. 1990). Patients with PCa appear to have higher circulating and organ levels of Cd (Brys et al. 1997).

Cd exposure has been reported to cause neoplastic transformation of human prostatic epithelial cells; however, the efficacy of this transformation is highly dependent on the dose of the metal ion (Achanzar et al. 2001; Nakamura et al. 2002). Exposure of normal human prostate epithelial cells to 10 μM Cd transiently increased the expression of *p53*, *c-myc*, and *c-jun* after 2 hr as a prelude to apoptosis (Achanzar et al. 2000). Longer exposure to 10 μM Cd resulted in the emergence of malignant phenotypes, including resistance to apoptosis, increased cell proliferation rate, disruption in DNA repair mechanisms, broad-based changes in gene expression, and epigenetic alterations (Goyer et al. 2004).

We are particularly interested in the contribution of noncytotoxic, low doses (< 10 μM) of Cd to neoplastic transformation of human prostate epithelial cells because of the relevance to environmental health. In this study we investigated the effects of low, noncytotoxic doses of Cd on the growth, cell-cycle distribution, and gene expression of an immortalized human normal prostate epithelial cell line (NPrEC). We found that low doses of Cd promoted growth in NPrEC cells and that 2.5 μM Cd induced overexpression of a set of genes all linked to tumor necrosis factor-α [*TNF*α; Entrez Gene ID 7124 (National Center for Biotechnology Information 2008a)], which exhibited a transient but drastic up-regulation after Cd exposure. These genes are related primarily to inflammation, immunomodulation, and oncogenesis. These findings suggested that Cd can directly trigger a proinflammatory/pro-oncogenic response in normal prostatic epithelial cells in the absence of paracrine signals from the stroma.

## Materials and Methods

### Cell culture

The NPrEC cell line, which shows a basal epithelial cell phenotype, was established in our laboratory (Mobley et al. 2003). The cells were grown in Defined Keratinocyte-SFM medium (Invitrogen, Carlsbad, CA) with growth-promoting supplement. Cell cultures were maintained at 37°C in a humidified incubator with a 5% CO_2_ atmosphere.

### Cell-viability assay

We seeded 5 × 10^3^ NPrEC cells in each well of a 96-well plate in quadruplicate. After 72 hr, the medium was replaced with 200 μL of fresh medium containing 0, 1, 2.5, 5, 10, or 20 μM cadmium chloride. Cell viability was determined after 24, 48, and 72 hr of treatment by the CellTiter 96 Aqueous One Solution Cell Proliferation Assay [3-(4,5-dimethylthiazol-2-yl)-5-(3-carboxymethoxyphenyl)-2-(4-sulfophenyl)-2H-tetrazolium (MTS)] kit (Promega, Madison, WI).

### Cell-cycle analysis

NPrEC cells were seeded at 8 × 10^5^ cells per 75-cm^2^ flask and synchronized by maintaining the cells in medium without supplement for 72 hr. The medium was then replaced with medium that included the supplement to induce synchronized growth (0 hr time point) and then treated or not treated with 2.5 μM CdCl_2_ for 4, 8, 16, or 32 hr. Flow cytometry was performed twice as described previously (Wetherill et al. 2002).

### RNA isolation

We extracted total RNA from NPrEC cells with TRIzol reagent (Invitrogen) according to the manufacturer’s instructions. RNA quality was assessed by the absorbance ratio at 260/280 nm and gel electrophoresis before further analysis.

### Global transcriptional profiling

We performed global transcriptional analysis using the Human Expression Array U133 Plus 2.0 arrays (Affymetrix, Santa Clara, CA), which have 54,675 probe sets. Sample preparation for array hybridization was carried out with One-Cycle Target Labeling and Control Reagents (Affymetrix). After fragmentation, the biotinylated cRNA was hybridized to arrays at 45°C for 16 hr. The arrays were then washed, stained with streptavidin-phycoerythrin, and scanned with a probe array scanner. Images of the scanned chips were analyzed with the Affymetrix GeneChip Operating System. Hybridization intensity data were converted into a presence/absence/marginal call for each gene, and changes in gene expression between experiments were detected by comparison analysis.

### Transcriptome data analyses

The data reported here have been deposited in NCBIs Gene Expression Omnibus (Barrett et al. 2006) and are accessible through accession no. GSE9951 (National Center for Biotechnology Information 2008b). Microarray analyses were performed in replicates for each of the five time points (0, 4, 8, 16, 32 hr) with Cd treatment and a no-Cd control. A total of 20 microarrays were used. The data were analyzed to identify genes whose expression was altered by Cd treatment at each of four time points (4, 8, 16, and 32 hr) compared with the zero time point. Analysis was performed with R statistical software (R Foundation for Statistical Computing 2008) and the LIMMA package for the Bioconductor (Smyth 2004). We used the rate monotonic algorithm to perform all steps of data preprocessing, including background correction, normalization, and expression set summaries. Chip quality was assessed with the affyQCReport package (Bioconductor 2008). One chip (Cd treatment at 4 hr) was removed from the analysis because of poor quality. Estimated fold changes at each time point were calculated by one-way analysis of variance (ANOVA), and resulting *t*-statistics from each comparison were modified by an intensity-based empirical Bayes method (Sartor et al. 2006). Genes for which all non-zero time points had a false discovery rate < 0.05 were examined according the fold change of the gene expression in the four nonzero time points (Table 1). The results were further scrutinized according to gene ontology, biological processes, molecular function, and genetic networks with the aid of Ingenuity Pathways Analysis (IPA; Ingenuity Systems, Mountain View, CA). IPA software maps the biological relationship of uploaded genes into networks according to published literature in the database. A relevancy score is assigned to each network in the data set to estimate the relevancy of the network to the gene list uploaded. A higher relevancy score means that the network is more relevant to the gene list entered. We selected the three highest scored networks; genes in these networks were selected for further post hoc analyses. Top pathways in each network, if available, were listed according to their *p*-values.

### Neutralization of TNF

We used purified monoclonal TNF neutralization antibody (TNF Ab, Clone 1825; R&D Systems, Minneapolis, MN) to neutralize the biological activity of TNF. TNF is a multifunctional proinflammatory cytokine secreted from the cells, which functions through its receptors. In addition to Cd treatment, another panel of the cells was co-treated with 4 μg/mL TNF Ab.

### Real-time reverse transcriptase-polymerase chain reaction (RT-PCR)

All primer pairs were designed to cross at least one intron (Table 2). Reverse transcription was performed using SuperScript III (Invitrogen) with 0.5 μg RNA per 20 μL of reaction mixture. For real-time PCR, we used the Power Sybr Green kit (ABI, Foster City, CA) in a 7500 Fast Real-Time System (ABI) in standard mode. A total of 0.5 μL cDNA was added to a 20 μL reaction. We used *GAPDH* and *18S* rRNA as the internal control, as described previously (Zhang et al. 2007), and found similar results (data not shown). Real-time RT-PCRs were performed in quadruplicate and independently repeated twice with two sets of cell cultures different from those used in the microarray. We used the 2^−ΔΔCt^ method with the tested primers to calculate relative expression levels of the transcripts; the efficiencies for the various real-time PCRs were determined to be close to 100%.

### In silico *analyses*

We retreived the sequences of the genes from Entrez Gene (National Center for Biotechnology Information 2008a), and information regarding their genomic organization was obtained by a BLAT search (UCSC Genome Bioinformatics 2008). Primers were designed with Primer3 (Table 2). Information on the genes are listed in Table 1.

### Statistical analysis

We performed two-way ANOVA with a Bonferroni post hoc test on data obtained from the MTS assays, cell-cycle analyses, and real-time RT-PCR quantification of relative transcript levels. We considered a *p* < 0.05 statistically significant.

## Results

### Low-dose CdCl_2_ exposure increases cell viability

**Figure 1:**
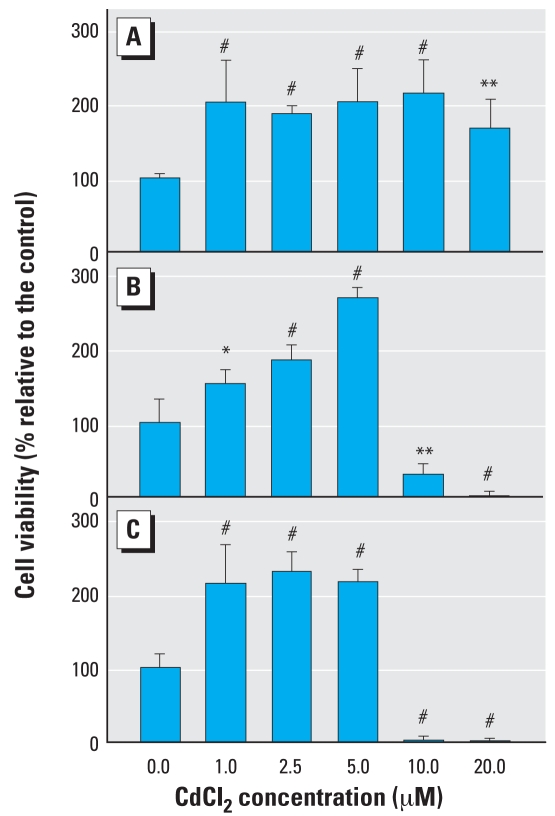
Effect of CdCl_2_ on viability of NPrEC cells treated with different concentrations of CdCl_2_ for 24 (*A*), 48 (*B*), or 72 (*C*) hr. Cell viability was determined by MTS assay. Each time point represents the mean value of quadruplicates ± SD. *p < 0.05. **p < 0.01. ^#^*p <* 0.001.

The effect of CdCl_2_ concentrations on the viability of NPrEC cells was evaluated at different time points (Figure 1). Compared with the viability of the control with no Cd treatment, which was set as 100%, cell viability was increased 150–270% after 24, 28, and 72 hr of treatment with 1, 2, 5, or 5 μM CdCl_2_. These increases could be due to a promotion of cell growth. However, the viability of cells exposed to 10 or 20 μM CdCl_2_ was enhanced 170–240% during the first 24 hr, followed by a dramatic loss of cells (> 70%) after 48 hr, and the death of almost all cells after 72 hr (~ 98%). Thus, concentrations of Cd ≥ 10 μM were cytotoxic to NPrEC cells. Treatment of NPrEC cells with 1, 2.5, or 5 μM CdCl_2_ for 3 weeks did not elicit a cytotoxic response. Compared with the viability in controls with no Cd treatment, cell viability in the 1 μM and 2.5 μM Cd-treated cell cultures exhibited modest increases (~ 20%) in cell viability (data not shown), but no change in cell viability was observed in cultures exposed to 5 μM Cd compared with controls. Based on these data, we used the noncytotoxic, growth-promoting concentration of 2.5 μM CdCl_2_ for subsequent experiments.

### Biphasic effects of Cd in cell cycle progression

**Figure 2:**
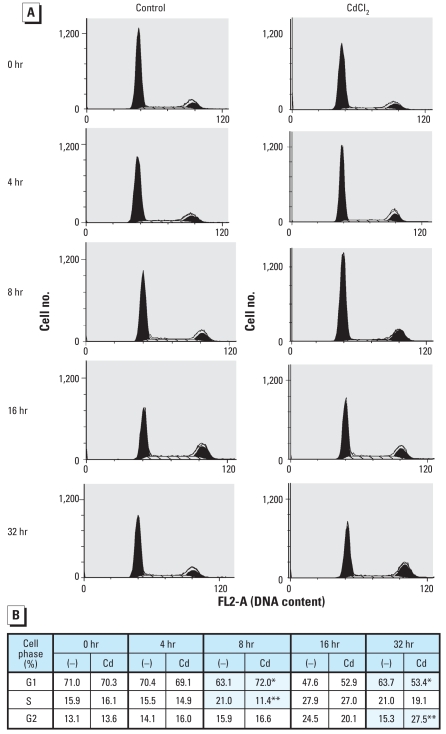
Effect of CdCl_2_ on cell-cycle distribution in NPrEC cells determined by flow cytometry analysis. (*A*) Fluorescence analysis of the DNA content. (*B*) Cd-induced change of the cell phase. The sub-G1 peak, an indication of apoptosis, is not shown. Fluorescence-2 area (FL2-A) is a measure of integrated cell fluorescence signal that represents the DNA content. Data represent results from two replicates. *p < 0.05. **p < 0.01.

We evaluated the effect of 2.5 μM CdCl_2_ on cell-cycle distribution of NPrEC cells after cells were synchronized by supplement deprivation for 72 hr (Figure 2). The synchronization technique reduced the background noise in cell cycle analyses but was not expected to affect cell growth or death induced by the Cd treatment per se. Compared with the control cells with no Cd treatment, cells exposed to Cd for 8 hr showed an increase in the G1 phase (from 63.1% to 72.0%) and a reduction in cells in the S phase (from 21.0% to 11.4%) (Figure 2). However, cells treated longer (32 hr) progressed through the cell cycle faster than did the control, resulting in an increase in cells in the G2 phase (27.5% of treated cells vs. 15.3% of control) and a decrease in cells in the G1 phase (63.7% of the treated cells vs. 53.4%). Our flow cytometry data indicated a transient blockage of cell-cycle progression at 8 hr, followed by acceleration after NPrEC cells were exposed to Cd 32 hr. Notably, the sub-G1 peak, an indication of apoptosis, is not evident in Figure 2.

### Transcriptome and gene ontology analyses

**Figure 3:**
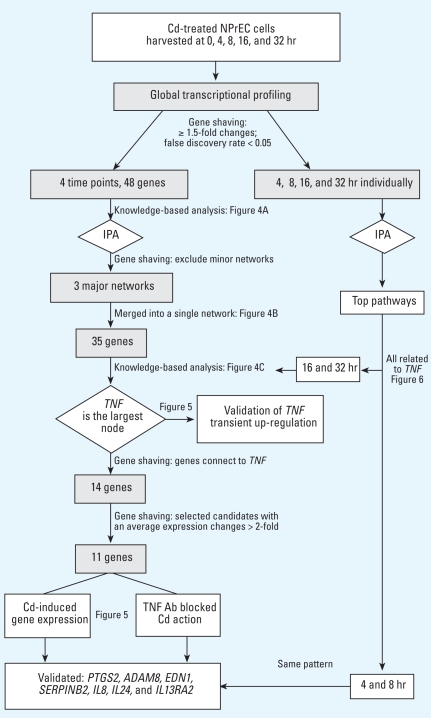
A schematic diagram illustrating the strategies and approaches used in candidate identification, gene shaving, knowledge-based analysis, and validation of a Cd-induced, *TNF*-regulated transcriptome in NPrEC cells.

**Figure 4:**
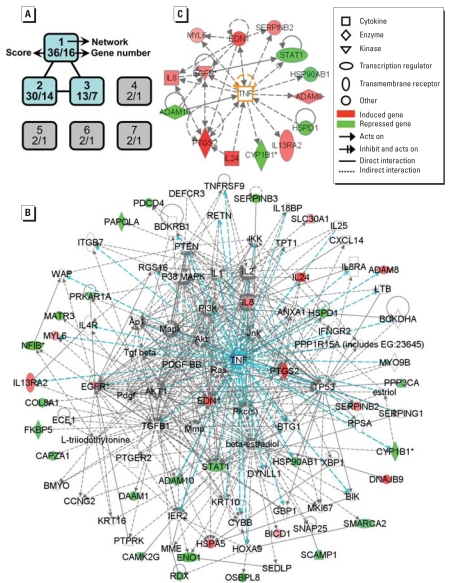
Pathway analysis of genes differentially expressed by microarray in Cd-treated NPrEC cells. When the 48 genes (Figure 3, Table 1) were input into IPA, it mapped them to three networks with high relevancy scores and four networks of low scores (*A*). The three high score networks were merged into a single network with 35 genes (*B*), with *TNF* as the largest node connected to 14 genes (*C*). The score indicates the degree of relevance of a network to the molecules in the input data set, which takes into account the number of network-eligible genes and the size of the network. Additional information is available at the IPA website (Ingenuity Systems 2008). The brighter the color of the gene, the higher the fold changes. *Multiple identifiers in the array data set file map to a single gene.

We assessed the effects of CdCl_2_ on changes in gene expression at 4, 8, 16, and 32 hr after exposure to Cd by global transcriptional profiling using a whole genome array with 54,675 probe sets (Figure 3). Forty-eight known genes (excluding three duplicate genes, two hypothetical genes, and two unknown genes) were differentially expressed in the control and Cd-treated cultures for all four time points investigated in the microarray data (Table 1). This initial “cutoff” criterion was chosen based on our experiences (Syed et al. 2005; Tam et al. 2008); changes in gene expression < 1.5-fold are difficult to be validated by real-time RT-PCR. We conducted gene ontology analyses on these Cd-targeted genes by IPA (input: 48 genes). Genes were mapped principally to three major networks (Figure 4A) with the highest relevancy scores: *a*) cardiovascular system development and function, cellular movement, and cancer; *b*) cellular growth and proliferation, hair and skin development and function, and cell cycle; and *c*) immunologic disease, inflammatory disease, and tissue morphology. Because of overlaps of the three networks, we used IPA to merge them to a larger network containing 35 of the original 48 genes (Figure 4B).

### Validation of transcriptome profiling data

**Figure 5:**
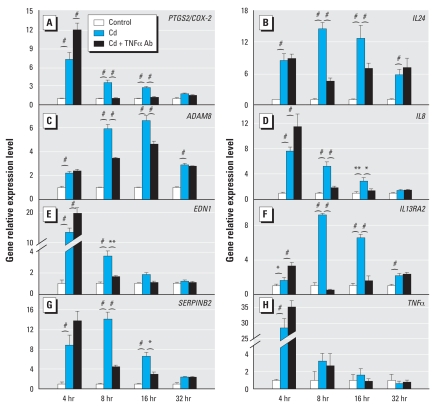
Validation of microarray data and investigation of the role of *TNF* in Cd-treated NPrEC cells. Real-time RT-PCR confirmed the up-regulation of 7 of the 11 *TNF*-related genes inferred by IPA. It also demonstrated a transient increase in *TNF* mRNA expression following Cd treatment. Each time point represents the mean value of quadruplicates ± SD. Two-way ANOVA compared Cd-treated group with control and Cd + Ab group, respectively. *p < 0.05. **p < 0.01. ^#^*p* < 0.001.

Fourteen genes were identified by IPA to have a known connection to *TNF* (Figure 4C). Eleven of them exhibited an average of ≥ 2-fold change in microarray signals for four time points following Cd-treatment (Table 1, footnote *c*). Real-time RT-PCR confirmed that Cd induced an up-regulation of prostaglandin-endoperoxide synthase 2 (*COX-2*/*PTGS2*), ADAM metallo-peptidase domain 8 (*ADAM8*), endothelin 1 (*EDN1*), serpin peptidase inhibitor, clade B (ovalbumin), member 2 (*SERPINB2*), interleukin 24 (*IL24*), *IL8*, and interleukin 13 receptor, alpha 2 (*IL13RA2*) (Figure 5) at most time points. Of the 28 pairs of comparison groups (control and Cd-treated, 7 genes at four time points; total of 56 groups), 23 pairs of comparison groups (82%) exhibited differences at a significance of *p* < 0.05 and 21 groups (75%) at *p* < 0.001. This demonstrated a high degree of concordance between the microarray data and the quantification by real-time RT-PCR. Cd also induced a down-regulation of cytochrome P450B1 (*CYP1B1*) *ADAM10*, *HSPD1*, and *STAT1*. Real-time RT-PCR validated the down-regulation of these genes at two time points (data not shown). Furthermore, among the genes shown in Figure 4B, we had picked three genes—*SERPINB3, HSPA5,* and *DNAJB9*—for real-time PCR validation and were able to confirm same direction of change at three time points as the microarray data (data not shown). The latter finding further demonstrated the effectiveness of identification of gene/network by global transcription profiling combined with knowledge-based analyses.

### TNF plays a central role in Cd-induced alteration of gene expression

To determine if TNF mediates the action of Cd in regulating the genes in the demonstrated network, we first showed a 28-fold transient increase in the accumulation of *TNF* transcripts after 4 hr of Cd exposure (Figure 5). We then co-treated NPrEC cultures with Cd plus TNF Ab and observed significant blockade of the Cd-induced up-regulation of all seven genes at most time points following the co-treatment. Of the 14 pairs of comparison groups (28 individual groups; Cd and Cd + TNF Ab) at 8 and 16 hr, significant blockade of the Cd-induced gene alteration by TNF Ab was exhibited in 13 pairs of comparison groups (93%) at *p* < 0.05 and 10 groups (71%) at *p* < 0.001. At 32 hr, however, we observed no significant differences between the Cd-treated and the Cd + TNF Ab–treated cultures, which is consistent with the finding of no significant increase in TNF transcripts in Cd-treated cultures at this late stage. However, the down-regulation of *CYP1B1* by Cd exposure was not reversed by the addition of TNF Ab to the culture medium (data not shown).

### Microarray and pathway analysis at individual time points

**Figure 6:**
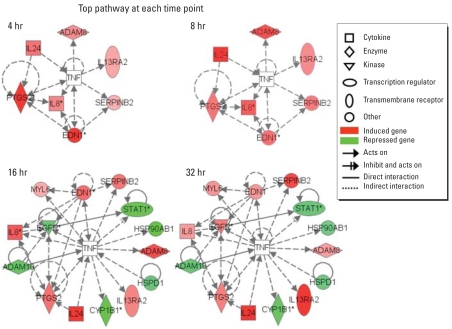
Pathway analysis of genes differentially expressed at each of the four time points. At 4 and 8 hr of Cd treatment, genes exhibited in the top pathway were the same eight genes confirmed by real-time RT-PCR (Figure 5). With longer Cd exposure at 16 and 32 hr, the top pathway exhibited the same pattern as shown in Figure 4C. The brighter the color of the gene, the higher the fold changes.

We were concerned that we may have lost valuable information because of our initial gene-shaving strategy of including only genes that displayed ≥ 1.5-fold change across all four time points. To address this concern, we reanalyzed the microarray data in a different manner. We identified genes affected by Cd treatment (≥ 1.5-fold changes and false discovery rate < 0.05) at each time point: for 4, 8, 16, and 32 hr of Cd treatment, we identified 2,211, 1,995, 1,871, and 1,087 genes, respectively. When these gene sets were individually analyzed with IPA, the top pathway identified for each of the four time points was invariably one that was connected to TNF (Figure 6). Importantly, we found the same seven genes to be connected to TNF at each of the four time points: *COX-2* /*PTGS2*, *ADAM8*, *EDN1*, *SERPINB2*, *IL24*, *IL8*, and *IL13RA2*. These were the same genes identified earlier using our initial gene-shaving strategies (Figure 3), and they were confirmed to be up-regulated by Cd and responsive to TNF-neutralizing antibody reversal (Figure 5). At 16 and 32 hr of Cd-treatment, an additional seven genes were found to be linked to TNF, yielding a total of 14 genes in the network. Interestingly, these 14 genes were identical to those shown in Figure 4C, which shows a network identified using the initial gene-shaving criteria. These findings collectively removed the concern of potential limitation of our initial gene-shaving strategy. Furthermore, they have strengthened our claim that the effect of Cd on NPrEC was mediated by TNF.

## Discussion

Unequivocally, Cd is a carcinogen for the rat prostate, but its oncogenic action on the human gland remains debatable (Waalkes 2003). Recent investigations have demonstrated that the metal ion could induce neoplastic transformation of human prostatic epithelial cells (Achanzar et al. 2001; Nakamura et al. 2002) that is accompanied by evasion of apoptosis (Qu et al. 2007). However, the mechanisms underlying the initiation of carcinogenesis by Cd in the human prostate are still not fully understood. Emerging evidence now indicates a strong association between chronic prostatic inflammation and human PCa (Sciarra et al. 2007). Cd is excreted at a rate of approximately 0.001%/day; therefore, it accumulates in the body for decades (Satarug and Moore 2004). An age-dependent increase in body burden of Cd and chronic exposure of the prostate to Cd may promote persistent inflammation, which is associated with increased cell proliferation and evasion of apoptosis, favoring neoplastic transformation in the prostate.

Immortalized normal prostate epithelial cell lines such as NPrEC are a useful model for the study of early events underlying prostate carcinogenesis. We exposed synchronized NPrEC cells to different concentrations of Cd and found that low levels of CdCl_2_ (≤ 5 μM) consistently increased cell viability but that higher levels inevitably led to cell demise with 72 hr of exposure. A similar biphasic response has been reported previously (Achanzar et al. 2000). The mitogenic response to low-dose Cd appeared to involve a transient blockage of cell-cycle progression at 8 hr, followed by acceleration through the cycle. These changes suggested major changes in the NPrEC expression transcriptome that might provide a mechanistic explanation for Cd-induced neoplastic transformation of normal prostate epithelial cells. With this rationale in mind, we exposed synchronized NPrEC cell cultures to a low-dose of Cd (2.5 μM) and investigated changes in global gene expression over time.

We used a stringent gene-shaving strategy coupled with knowledge-based analyses to uncover changes in gene expression most relevant to biological responses. We have identified, for the first time, that *TNF* is the most prominent node in a network of Cd-regulated genes related to immunomodulations, oncogenesis, cell proliferation, and apoptosis. Among the seven up-regulated genes identified to be linked to *TNF*, real-time RT-PCR validated three genes that were changed > 2-fold across at all four time points, three across three time points, and one at two time points. Furthermore, Cd exposure dramatically increased *TNF* expression (28-fold) during the early stage (4 hr); this in turn led to an up-regulation of seven genes in the later stage of response (8–16 hr). Most important, when we used a TNF-neutralizing antibody to negate the autocrine effects of the cytokine in NPrEC cultures, the up-regulation of seven genes by Cd was blocked, providing strong evidence that these genes are downstream targets of TNF. Anti-TNF monoclonal antibodies have also been used in patients for anti-TNF treatment (Shealy and Visvanathan 2008). It should be noted that our initial analyses of the microarray data did not identify *TNF* as a target gene whose expression was significantly altered by Cd. In this regard, knowledge-based analysis has certainly added a new strategic dimension to the analysis of microarray data. These findings collectively illustrated a high degree of validity of using a combined approach of global transcription profiling and knowledge-based analysis for gene network discovery.

Although Cd cannot form stable DNA adducts and is not a redox-active metal (Waalkes 2000), the induction of *TNF* and its downstream target genes could lead to mutagenic changes necessary for the development of epithelial cancers (Babbar and Casero 2006). TNF is a cytokine involved in systemic inflammation with a primary role of regulating immune cells. For instance, exposure of human bronchial epithelial cells to TNF was found to increase intracellular reactive oxygen species via an induction of spermine oxidase and to lead to oxidative DNA damage, as indicated by the accumulation of 8-oxo-deoxyguanosine in cell nuclei (Babbar and Casero 2006). If a parallel could be drawn for NPrEC, Cd-induced overexpression of TNF and its associated autocrine signaling could lead to the mutagenic changes necessary for neoplastic transformation.

Of the seven TNF-up-regulated genes identified, PTGS2 (COX-2) is involved in inflammation-mediated oxidative stress favoring prostatic carcinogenesis (Tam et al. 2007). This enzyme is overexpressed in human prostate adenocarcinoma, and its inhibitors hold promise for PCa prevention and therapy (Hussain et al. 2003). ADAM8 is a catalytically active metallo-proteinase with a purported role in the degradation of the vascular basement membrane (Handsley and Edwards 2005). The over-expression of ADAM8 in PCa is associated with parameters of unfavorable prognosis (Fritzsche et al. 2006). EDN1, the most potent vaso-constrictor known, acts as a survival factor for endothelial cells. Within the prostate, EDN1 is mainly epithelial, while its receptors are present in the stroma and epithelium. EDN1 is elevated in the plasma of patients with hormone-refractory PCa and stimulates osteoblastic remodeling, suggesting a role in the development of bone metastases (Granchi et al. 2001). EDN1 is suspected to act as an autocrine factor during malignant transformation (Granchi et al. 2001). It is overexpressed in PCa and inhibits apoptosis (Godara et al. 2007). The up-regulation of EDN1 in Cd-treated NPrEC cells is consistent with the observation that no sub-G1 peak was observed in the flow cytometry result (Figure 2). IL8 is a powerful chemotactic factor that provides a growth advantage to tumor cells. In particular, IL8 expression in the prostate correlates positively with tumor progression and cell dedifferentiation (Lee et al. 2004), and its levels are higher in the serum of patients with metastatic PCa (Murphy et al. 2005). A parallel increase in IL8 and its receptors has been associated with proliferation and microvessel density in PCa. Thus, IL8 in the prostate have been deemed responsible for PCa initiation and promotion (Murphy et al. 2005). IL13RA2, one of the components of the type I IL13R, is frequently expressed on the surface of different cancer cells (Kawakami 2005). Expression of IL13RA2 is high in ovarian cancer but very low in the normal ovary (Kioi et al. 2006). IL13RA2 dramatically enhances the antitumor effect of IL13 receptor–targeted cytotoxin in human PCa xenografts (Kawakami et al. 2001). Meanwhile, no direct studies have reported a role of SERPINB2 and IL24 in PCa.

SERPINB2 has been shown to inhibit urokinase-type plasminogen activator, which is expressed at higher levels in PCa tissues (Wang and Jensen 1998); Delivery of IL24 to the cells profoundly inhibits PCa cell growth (Sarkar et al. 2007). The overexpression of *SERPINB2* and *IL24* may be an attempt by NPrEC cells to guard against the unfavorable Cd challenge.

We also conducted an exhaustive literature search and found that six of seven genes found to be connected to TNF by IPA analyses had previously been reported to be regulated by TNF at the promoter, transcript, or protein level: *PTGS2* (Chen et al. 2000; Ikawa et al. 2001; Subbarayan et al. 2001), *IL8* (Lora et al. 2005; Rathanaswami et al. 1993; Treede et al. 2007), *EDN1* (Woods et al. 2003), *ADAM8* (Schlomann et al. 2000; Banno et al. 2004), *IL13RA2* (David et al. 2003), and *SERPINB2* (Wang and Jensen 1998). These documented connections between TNF and genes identified in this study further solidify our belief of the existence of such a network in NPrEC cells.

In the first step in our initial gene-shaving scheme, the genes for knowledge-based analyses were limited to those that were significantly changed at all four treatment time points. This stringent criterion might filter out potentially valuable information due to the limited number of genes included. With this concern in mind, knowledge-based analyses were also performed using microarray data collected at individual time points. Using this approach, we consistently identified a TNF-network as the top network linking genes affected by Cd-treatment of NPrEC cells, and we found that the genes connected to this TNF-network were identical to the genes discovered using our initial criteria. These findings inspire confidence in our original strategy scheme for data analysis and lend credence to our claim that TNF is an early mediator of Cd-action on NPrEC cells.

In summary, using NPrEC cells as a model, we have identified for the first time a TNF-associated network that is responsive to low-dose Cd exposure. Genes in this network are involved primarily in inflammation and immunomodulation that are linked to carcinogenesis. Identification of this regulatory pathway has shed new light on the mechanism of Cd-mediated prostate carcinogenesis that may involve a transient, “intrinsic” overexpression of *TNF* in the prostatic epithelium. Finally, this study represents one of those rare success in which global transcriptional profiling was able to formulate a novel hypothesis that was subsequently tested.

## Figures and Tables

**Table 1:** Genes expressed differentially at four time points in NPrEC cells treated with 2.5 μM CdCl_2_.

ID^a^	Gene	4 hr	8 hr	16 hr	32 hr	Mean	Network^b^
81611	*ANP32E*	−3.01	−3.56	−3.46	−2.32	−3.09	
6317	*SERPINB3*	−1.83	−5.35	−1.54	−1.74	−2.62	1
2023	*ENO1*	−1.91	−1.87	−4.20	−2.01	−2.50	2, 3
81688	*C6orf62*	−1.90	−2.52	−3.01	−1.84	−2.32	
102^c^	*ADAM10*	−2.24	−2.38	−2.82	−1.71	−2.29	1
9749	*PHACTR2*	−1.61	−3.03	−2.34	−2.13	−2.28	
9782	*MATR3*	−2.34	−2.53	−2.40	−1.81	−2.27	2
4781	*NFIB*	−2.09	−2.56	−2.64	−1.55	−2.21	1
114882	*OSBPL8*	−2.80	−2.12	−1.87	−1.97	−2.19	2
1545^c^	*CYP1B1*	−2.62	−2.31	−1.72	−1.99	−2.16	2
3329^c^	*HSPD1*	−2.53	−1.98	−2.48	−1.58	−2.14	1
89890	*KBTBD6*	−2.60	−2.10	−1.95	−1.66	−2.08	
10914	*PAPOLA*	−1.79	−1.99	−2.53	−1.78	−2.02	3
6772	*STAT1*	−1.75	−2.70	−2.00	−1.52	−1.99	1
55353	*LAPTM4B*	−1.52	−2.24	−2.59	−1.60	−1.99	
2289	*FKBP5*	−1.68	−2.57	−2.02	−1.67	−1.98	2
829	*CAPZA1*	−1.88	−1.85	−2.08	−1.99	−1.95	2
5530	*PPP3CA*	−1.84	−1.85	−1.83	−2.21	−1.93	3
55854	*LEREPO4*	−2.20	−2.22	−1.76	−1.50	−1.92	
23002	*DAAM1*	−1.97	−2.31	−1.75	−1.57	−1.90	2
11137	*PWP1*	−1.75	−2.29	−1.70	−1.65	−1.85	
55055	*ZWILCH*	−1.75	−1.99	−2.03	−1.60	−1.84	
9522	*SCAMP1*	−1.90	−2.06	−1.57	−1.76	−1.82	2
3326	*HSP90AB1*	−1.68	−1.93	−2.10	−1.51	−1.80	1
27250	*PDCD4*	−1.57	−2.25	−1.62	−1.74	−1.79	3
5962	*RDX*	−1.76	−1.69	−2.02	−1.69	−1.79	2
1295	*COL8A1*	−1.80	−1.90	−1.90	−1.50	−1.78	2
64710	*NUCKS1*	−1.62	−1.71	−1.96	−1.55	−1.71	
818	*CAMK2G*	−2.06	−1.59	−1.55	−1.52	−1.68	1
5573	*PRKAR1A*	−1.60	−1.57	−1.91	−1.56	−1.66	1
6595	*SMARCA2*	−1.68	−1.50	−1.67	−1.56	−1.60	2
636	*BICD1*	1.51	1.52	1.67	1.70	1.60	2
7779	*SLC30A1*	1.50	1.70	1.78	1.61	1.65	3
4637	*MYL6*	1.75	1.58	1.53	1.78	1.66	1
6991	*TCTE3*	1.70	1.64	1.66	1.96	1.74	
26776	*RNU71B*	1.90	1.71	2.12	1.92	1.91	
541466	*CT45-1*	2.21	1.82	1.58	2.05	1.91	
1956^c^	*EGFR*	2.27	1.92	2.77	2.17	2.28	1
5055^c^,^d^	*SERPINB2*	1.84	2.60	1.52	3.18	2.29	1, 3
3576^c^,^d^	*IL8*	2.89	3.20	1.86	1.72	2.42	1
101^c^,^d^	*ADAM8*	2.70	3.47	2.04	1.62	2.46	3
3598^c^,^d^	*IL13RA2*	2.14	2.31	2.18	3.56	2.55	3
3309	*HSPA5*	2.19	5.92	1.55	1.82	2.87	1, 2
1906^c^,^d^	*EDN1*	6.56	2.83	1.54	1.75	3.17	1
4189	*DNAJB9*	5.14	4.01	1.52	2.09	3.19	2
7718	*ZNF165*	6.28	3.48	2.04	1.55	3.34	
11009^c^,^d^	*IL24*	3.43	4.15	2.24	3.57	3.35	1
5743^c^,^d^	*PTGS2*	8.68	2.71	1.52	2.17	3.77	1

a This list includes 48 genes up-regulated or down-regulated by ≥ 1.5-fold at each time point, with a false discovery rate of < 0.05; IDs are from Entrez Gene (National Center for Biotechnology Information 2008a).

b Networks are as follows: 1, cardiovascular system development and function, development and function, cellular movement, cancer; 2, cellular growth and proliferation, hair and skin development and function, cell cycle; 3, immunologic disease, inflammatory disease, tissue morphology.

c Eleven genes were identified to have an average of > 2-fold change and directly linked to the *TNF* node in the merged three major networks.

d Seven of the 11 genes were validated by real-time RT-PCR at most time points in both Cd- and Cd + TNF Ab–treated groups.

**Table 2:** Primers used in real-time RT-PCR to validate microarray data.

Gene	Forward primer	Reverse primer	Size (bp)
*SERPINB2*	CACCCAGAACCTCTTCCTCTCC	TAACTGCATTGGCTCCCACTTC	134
*IL8*	CTCTTGGCAGCCTTCCTGATTT	TGGGGTGGAAAGGTTTGGAGTA	115
*ADAM8*	TGCTCCTCCGGTCACTGTGT	ACGTTGGCTTGATGACCTGCT	86
*IL13RA2*	TCTTGGAAACCTGGCATAGGTG	GCCTCCAAATAGGGAAATCTGC	146
*EDN1*	CTCTCTGCTGTTTGTGGCTTGC	GTGGACTGGGAGTGGGTTTCTC	107
*IL24*	CACACAGGCGGTTTCTGCTATT	AAGAATGTCCACTTCCCCAAGG	92
*PTGS2*	ATTCCCTTCCTTCGAAATGCAA	AGAGAAGGCTTCCCAGCTTTTG	117
*TNF*	CTCTTCTGCCTGCTGCACTTTG	CAGCTTGAGGGTTTGCTACAACA	157
